# Maternal vascular endothelial growth factor receptor and interleukin levels in pregnant women with twin-twin transfusion syndrome

**DOI:** 10.7150/ijms.61014

**Published:** 2021-07-11

**Authors:** Nguyen Duy Anh, Phan Huyen Thuong, Nguyen Thi Sim, Tran Thi Phuong Thao, Luong Thi Lan Anh, Than Thi Thu Canh, Nguyen Van Dieu, Nguyen Duy Bac, Hoang Van Tong

**Affiliations:** 1 Hanoi Obstetrics and Gynecology Hospital; 2 Institute of Biomedicine and Pharmacy, Vietnam Military Medical University; 3 Hanoi Medical University

**Keywords:** Twin-twin transfusion syndrome, IL-1β, IL-6, IL-8, VEGF-R1 and VEGF-R2

## Abstract

Twin-twin transfusion syndrome (TTTS) is an unusual and serious condition that occurs in twin pregnancies when identical twins share a placenta but develop discordant amniotic fluid volumes. TTTS is associated with an increased risk of fetal death and birth defects if untreated. This study investigated the soluble levels of biomarkers including growth factors and interleukins in pregnant women with and without TTTS during pregnancy. We quantified plasma levels of VEGF-R1, VEGF-R2, IL-1β, IL-6 and IL-8 in twin pregnant women with (n=53) and without TTTS (n=72) and in women with single pregnancy (n=30) by ELISA and analyzed the association of maternal circulating biomarker levels with TTTS. Our results showed that maternal VEGF-R1 levels were significantly higher in twins compared to single pregnancy (*P*<0.05) and were decreased in the second trimester compared to the first trimester (*P* = 0.065, 0.019 and 0.072 for twins with and without TTTS and single pregnancy, respectively). VEGF-R2 levels had a trend to be lower in twins compared to single pregnancy. In addition, soluble VEGF-R1 and VEGF-R2 levels were significantly decreased while IL-6 levels were increased after surgical treatment with laser in twin pregnant women with TTTS (*P* = 0.016, 0.041 and 0.04, respectively). These results suggest that IL-6, VEGF-R1 and VEGF-R2 are involved in vascular regulation and stabilization in twin pregnancies and may contribute to the pathogenesis of TTTS and thus play a prognostic role in the surgical treatment of TTTS.

## Introduction

Twin pregnancy is one of the causes of perinatal pregnancy complications such as preterm birth, stillbirth, fetal growth abnormality, and especially twin-twin transfusion syndrome (TTTS) 1-3. TTTS is one of the most fatal perinatal complications with a mortality rate of up to 80-100%, and birth defects in 15-50% of surviving infants 4. TTTS occurs in about 5.5-17.5% of monochorionic twins 5 and 0.07% of all pregnancies with 2,800 cases in the United States annually 6. The pathogenesis of TTTS is due to an imbalance in the placental circulation system between the two fetuses that leads to a difference in the amount of fetal blood through placenta transfusion 7. Consequently, the fetus gradually reduces volume, anemia and growth restriction, and thus amniotic fluid. In contrast, the receiving fetus has a rapid increase in volume, polycythemia vera with cyclic overload and severe poly amniotic fluid 8.

Although the number of arterial-artery vessels and veins-intravenous vessels is not significantly different in fetuses with TTTS, a significant decrease in the number of arterial-artery vessels has been observed in fetuses with TTTS compared to those without TTTS 9. These findings suggest that the arterial-artery junction is an essential part of maintaining a pressure gradient between the two placental circulation systems in twin pregnancy 10. A study conducted on 126 cases of twins with severe TTTS found that there is always a one-way blood flow from fetal donor to recipient, leading to an imbalance of blood transfusion in arterial-arteries as a premise for the development of TTTS. In addition, it appears that the number and type of vascular connections in the placenta influence the development of TTTS 11. However, the cause and exact mechanism by which TTTS occurs are not fully understood.

Placental artery formation plays a crucial role in the formation and development of the fetus. The most important molecules among blood vessel formation and development factors are vascular endothelial growth factors (VEGF). The VEGF family includes VEGF-A, VEGF-B, VEGF-C, VEGF-D, and placental growth factor (PGF) 12. VEGFs activate angiogenesis by binding to two receptors, VEFGR-1 (Flt-1) and VEGFR-2 (Flk-1) 13. While the VEGFR-1 receptor reduces the proliferation and branching of endothelial cells and capillary network, the VEGFR-2 receptor is responsible for the proliferation of vascular cells, promoting vascular branching and maintaining endothelial cells 14. Previous studies have shown that VEGF activity is regulated by VEGR-R1 that acts as an endogenous inhibitor of VEGF through the VEGF-specific binding action of the soluble VEGF-R1 resulting in a decrease of VEGF bound to VEGF-R2 15. It has been reported that VEGF-R1 levels increase during pregnancy, but subsequently decrease after birth 16. The mutual functional role of VEFGR-1 and VEGFR-2 receptors are essential for the formation of placental blood vessels. However, the changes and functional role of soluble VEFGR-1 and VEGFR-2 levels during TTTS development are not clearly understood.

The metabolism of pregnant women with TTTS has been shown to be abnormal at mid-pregnancy and is associated with amniotic fluid production rates in the fetuses 17. A study showed that maternal interleukin-6 (IL-6) levels are directly related to twin blood transfusions in infants 18. Elevated IL-6 levels in maternal amniotic fluid are significantly associated with preterm delivery and require clinical attention in the prediction of preterm birth and patient care guidelines 19. In addition, IL-8 levels are associated with the risk of preterm birth, suggesting that IL-8 is a prognostic marker of preterm birth in twin patients 20. Nevertheless, the association of inflammatory interleukin with TTTS pathogenesis needs to be further investigated.

With an important role of VEFGR-1 and VEGFR-2 in blood vessel formation and fetal development and of interleukins in the pathogenesis of TTTS, we investigate the concentrations of soluble VEFGR-1, VEGFR-2, IL-1β, IL-6, and IL-8 in women with and without TTTS during pregnancy and TTTS patients before and after surgical treatment.

## Materials and Methods

### Study subjects

**Figure 1:**
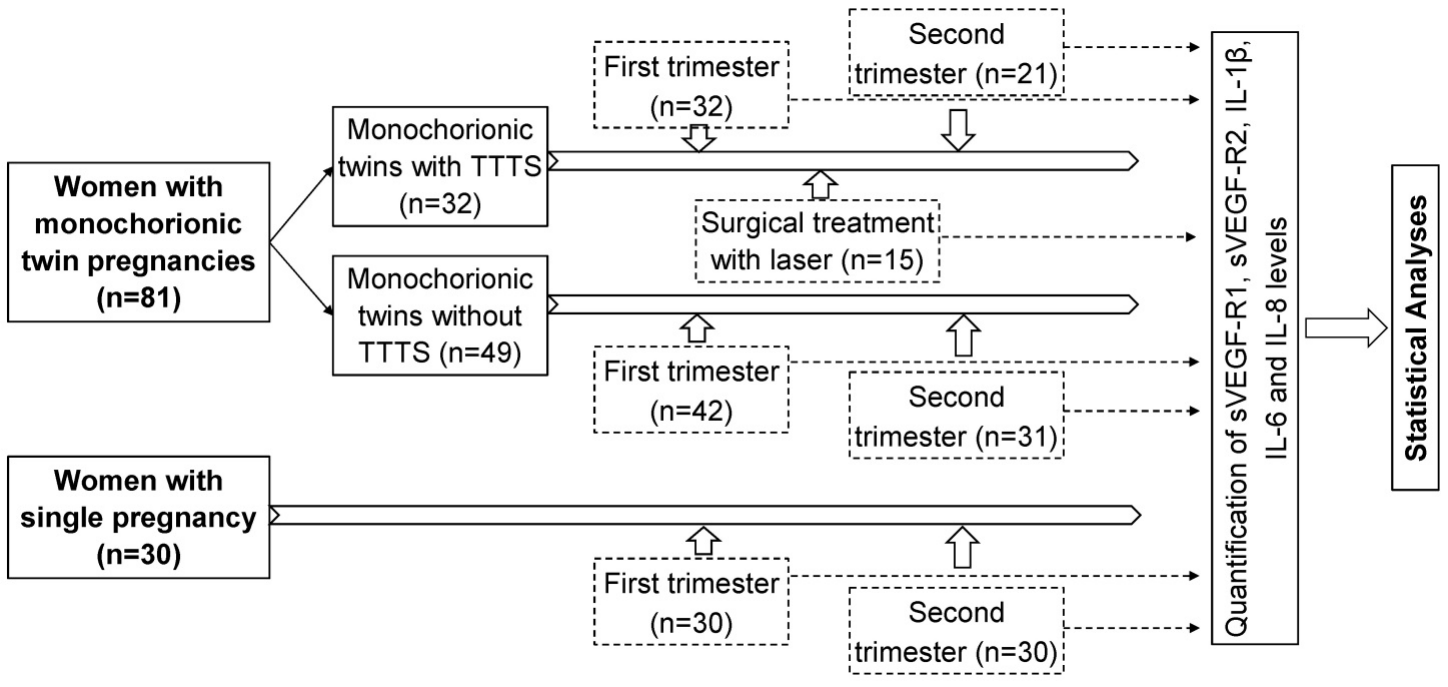
Strategy of investigation, sample sizes, and sampling timeline

A total of 81 women with monochorionic twin pregnancies admitted for regularly clinical examination during pregnancy at the Hanoi Obstetrics and Gynecology Hospital were recruited for this study. Monochorionic twin pregnancies were diagnosed by ultrasound according to the International Society of Ultrasound in Obstetrics and Gynecology (ISUOG) Practice Guidelines 21. The study subjects were divided into two main groups: monochorionic twins with TTTS (n=32), and those without TTTS as a control group (n=49). Among 32 monochorionic twins with TTTS, blood samples were collected at two points of time, the first trimester (n=32) and the second trimester (n=21). Among 49 monochorionic twins without TTTS, blood samples were collected in the first trimester (n=42) and the second trimester (n=31). Among 32 monochorionic twin pregnant women with TTTS, 15 were treated with laser surgery and blood samples were additionally collected one week after surgical treatment (n=15). The baseline characteristics and laboratory parameters of twin pregnant women with and without TTTS in the first trimester were presented in table 1 (Table 1). There was no significant difference in baseline laboratory parameters between twin pregnant women with and without TTTS except liver enzyme levels (p<0.05). In addition, 30 women with single pregnancy were also included as another control group and blood samples were collected at the first and second trimester. The strategy of investigation, sample sizes, and sampling timeline is presented in figure 1 (Figure 1). Peripheral blood samples from all participants were collected and centrifuged to separate plasma and were stored at -80ºC before use.

### Ethics statement

Pregnant women were informed in detail about the study and written consent was obtained from all participants. Procedures for taking samples and taking care of study participants were implemented according to the regular routines of the Hanoi Obstetrics and Gynecology Hospital. The study was approved by the Institutional Review Board of the Hanoi Obstetrics and Gynecology Hospital, Hanoi, Vietnam.

### Quantification of plasma VEGF-R1 and VEGF-R2 concentrations by ELISA

The concentrations of VEGF-R1 and VEGF-R2 were quantified in the plasma samples from study subjects by ELISA method using the VEGF Receptor 1 (Soluble) Human ELISA Kit (Invitrogen, Waltham, Massachusetts, USA, Catalog Numbers: BMS2019) and the VEGF Receptor 2 / KDR Human ELISA Kit (Invitrogen, Waltham, Massachusetts, USA, Catalog Numbers: BMS268-3), respectively. The procedures were followed according to the manufacturer's instructions.

### Quantification of Interleukin levels by ELISA

The levels of IL-1β, IL-6 and IL-8 were quantified in the plasma samples from study subjects by ELISA method using the Human IL-1β Instant ELISA Kit (Invitrogen, Waltham, Massachusetts, USA, Catalog Numbers: BMS224INST) and the Human IL-6 Instant ELISA Kit (Invitrogen, Waltham, Massachusetts, USA, Catalog Numbers: BMS213INST), and the Human IL-8 Instant ELISA Kit (Invitrogen, Waltham, Massachusetts, USA, Catalog Numbers: BMS204-3INST), respectively. The procedures were followed according to the manufacturer's instructions.

### Statistical analyses

All data were managed using Microsoft Excel 2013 and analyzed using SPSS v.20 software (IBM, Armonk, NY, USA). Clinical and demographic data were presented as median with range for continuous variables and as numbers with percentages for categorical variables. The student's *t*-test, one-way ANOVA, Kruskal-Wallis, Mann-Whitney *U* tests or Wilcoxon signed-rank tests were used to compared continuous variables including the levels of VEFGR-1, VEGFR-2, IL-1, IL-6, and IL-8, where appropriate. The Chi-square test or Fisher's exact test was used to compare categorical variables. Spearman's rank correlation coefficient was used to analyze the correlation between two variables and between those markers and clinical parameters. The level of significance was set at a *P*-value of less than 0.05.

## Results

### VEGF-R1 and VEGF-R2 concentrations in monochorionic twin pregnancy

**Figure 2:**
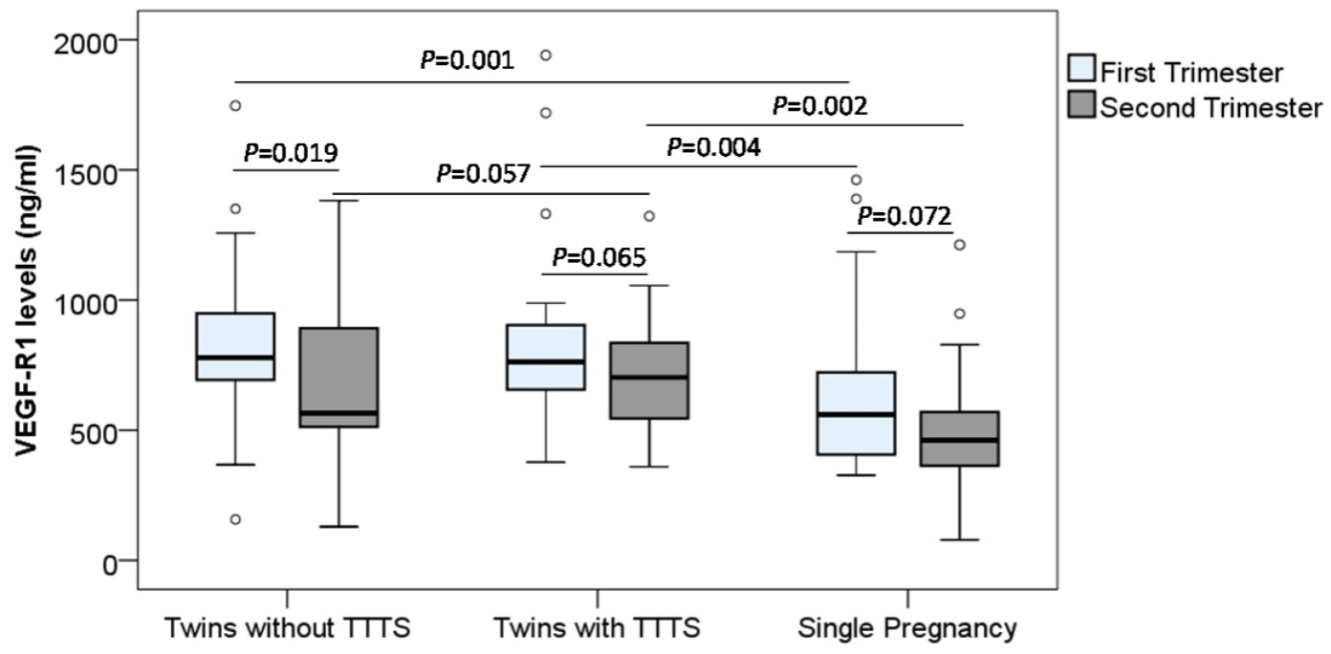
** Maternal soluble VEGF-R1 levels in pregnant women.** Soluble VEGF-R1 levels were measured in different subgroups of pregnant women. TTTS: Twin-to-twin transfusion syndrome; *P* values were presented reflecting the comparison between groups. Two-sided Mann-Whitney U test or Wilcoxon signed-rank test was used to compare between groups.

Soluble VEGF-R1 and VEGF-R2 concentrations were determined in monochorionic twin pregnant women with and without TTTS and women with single pregnancy. In the first trimester, we observed that soluble VEGF-R1 levels were significantly higher in monochorionic twin pregnant women with and without TTTS [median (min-max): 763.3 (376.9-1940.6) and 779.2 (157.1-1746.5), respectively] compared to those in single pregnant women [median (min-max): 560.5 (326.9-1461.6)] (*P*=0.004 and 0.001, respectively). In the second trimester, soluble VEGF-R1 levels in monochorionic twin pregnant women with TTTS were significantly higher compared to those in single pregnant women [median (min-max): 702.6 (359.5-1322.1) vs. 461.2 (78.6-1211.9)] (P=0.002). However, there was no significant difference in soluble VEGF-R1 levels between monochorionic twin pregnant women without TTTS and single pregnant women. Soluble VEGF-R1 levels were slightly increased in twin pregnant women with TTTS compared to those without TTTS [median (min-max): 702.6 (359.5-1322.1) vs. 565.5 (128.9-1381.7)] (P=0.057) (Figure 2).

**Figure 3:**
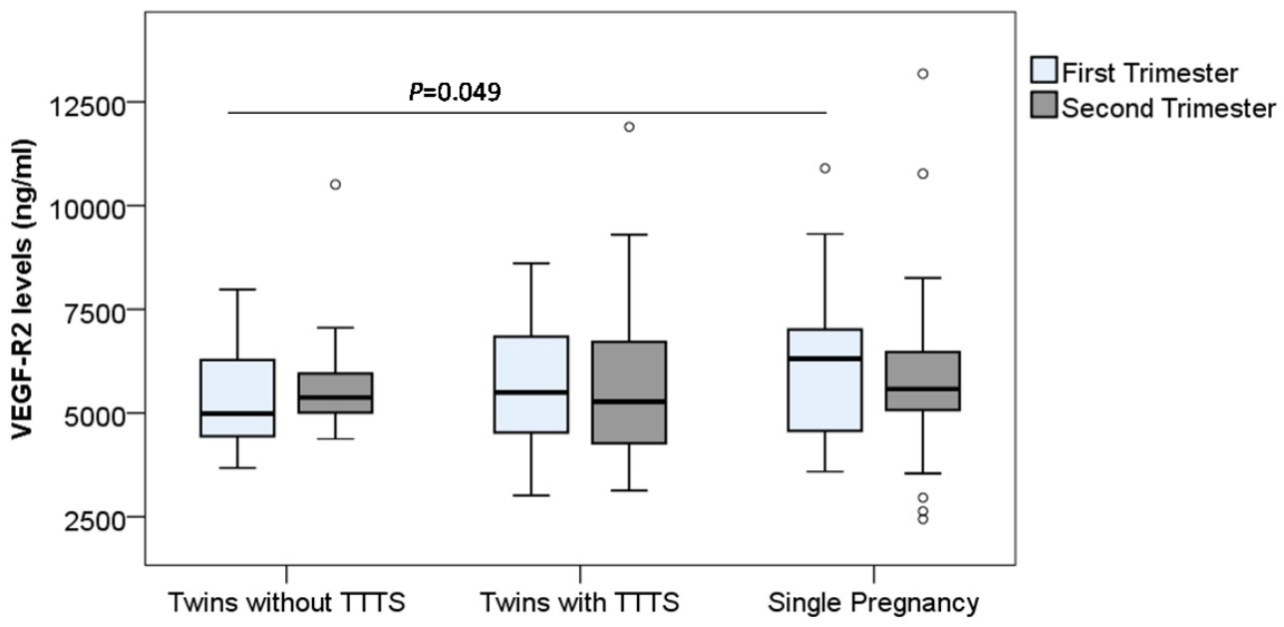
** Maternal soluble VEGF-R2 levels in pregnant women.** Soluble VEGF-R2 levels were measured in different subgroups of pregnant women. TTTS: Twin-to-twin transfusion syndrome; *P* values were presented reflecting the comparison between groups. Two-sided Mann-Whitney U test or Wilcoxon signed-rank test was used to compare between groups.

In contrast to the VEGF-R1 levels, we observed a trend that the VEGF-R2 levels in monochorionic twin pregnant women (both with and without TTTS) were lower compared to those in single pregnant women. In particular, the VEGF-R2 levels in monochorionic twin pregnant women without TTTS in the first trimester were significantly lower compared to those in single pregnant women [median (min-max): 4981.8 (3676.7-7977.9) vs. 6316.04 (3585.7-10899.7)] (P=0.049) (Figure 3). We compared the soluble VEGF-R2 levels between twin pregnant women with TTTS and without TTTS, but no significant difference was observed.

In order to examine the changes of soluble VEGF-R1 and VEGF-R2 levels during pregnancy, we compared their levels between the first and second trimesters, and we observed the trend that the VEGF-R1 levels were decreased in the second trimester compared to the first trimester. In particular, the difference of the VEGF-R1 levels between the first and second trimesters in the twin pregnant women without TTTS reached statistical significance (*P*=0.019) (Figure 2 and Figure 3). We also analyzed the correlation of VEGF-R1 and VEGF-R2 levels with laboratory parameters, however, the results did not reach statistical significance.

### VEGF-R1 and VEGF-R2 concentrations and surgical treatment

**Figure 4:**
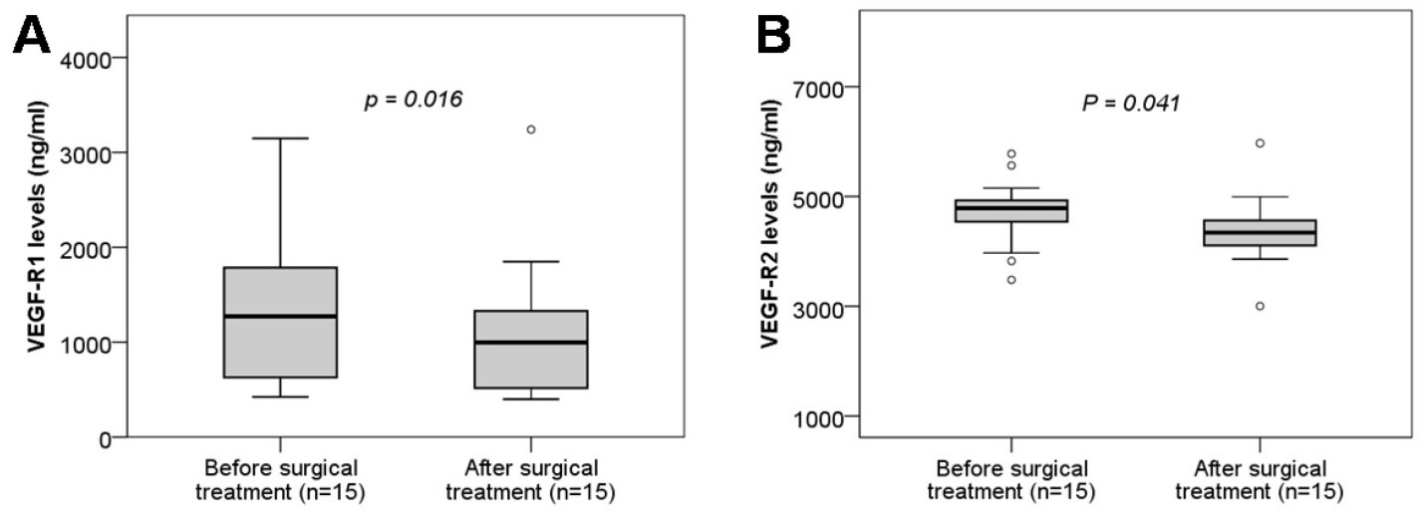
** Maternal soluble VEGF-R1 and VEGF-R2 levels in twin pregnant women with TTTS before and after surgical treatment.** Soluble VEGF-R1 and VEGF-R2 levels were measured in twin pregnant women with TTTS who were treated with surgery and were compared between before and after surgical treatment. TTTS: Twin-to-twin transfusion syndrome; *P* values calculated by Wilcoxon signed-rank test were presented reflecting the comparison between groups.

We analyzed the soluble VEGF-R1 and VEGF-R2 levels in twin pregnant women with TTTS who were treated with surgery by comparing soluble VEGF-R1 and VEGF-R2 levels between before and after surgical treatment. We observed that soluble VEGF-R1 and VEGF-R2 levels were significantly decreased after surgical treatment compared to those before surgical treatment (*P*=0.016, and 0.041, respectively) (Figure 4). These results indicated that surgical treatment strongly affects the development of the placental and fetal vascular system.

### Interleukin levels in monochorionic twin pregnancy

**Figure 5:**
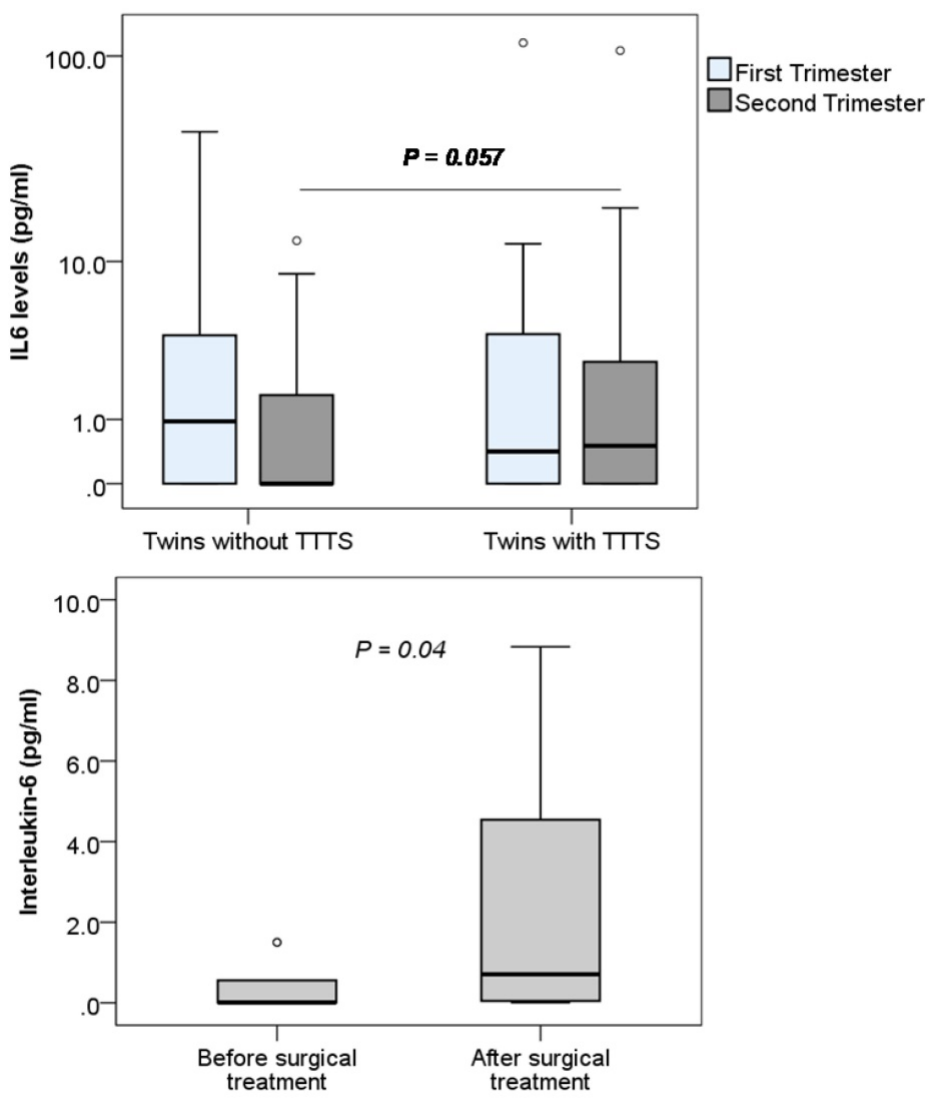
** Maternal Interleukin-6 levels in pregnant women.** Interleukin-6 levels were measured in different subgroups of pregnant women. TTTS: Twin-to-twin transfusion syndrome; *P* values were presented reflecting the comparison between groups. Two-sided Mann-Whitney U test was used to compare between groups.

The levels of IL-1β, IL-6 and IL-8 were determined in monochorionic twin pregnant women with and without TTTS and women with single pregnancy. We observed that the levels of IL-1β and IL-8 were below the detection limit of the ELISA kit (<7.8 pg/mL for IL-1β and <15.6 pg/mL for IL8) (data not shown). We could only detect IL-6 in the plasma samples of study pregnant women and compare between twin pregnant women with and without TTTS. We observed that maternal IL-6 levels were not significantly different between twin pregnant women with and without TTTS. Nevertheless, when data were segregated according to trimester, maternal IL-6 levels were marginally increased in twin pregnant women with TTTS compared to those without TTTS in the second trimester (*P*=0.057) (Figure 5). We also compared IL-6 levels in the twin pregnant women with TTTS who were treated with surgery. We observed that IL-6 levels were significantly increased after surgical treatment compared to those before surgical treatment (*P*=0.04).

## Discussion

Although the pathophysiology of TTTS has been known as an imbalance in supplying the blood of the two fetuses through the vascular junctions on the placenta, the cause of TTTS has not been fully understood. 22. It has been documented that the number, type and size of vascular junctions are key factors for the blood transfusion stability of monochorionic twins 11. Almost all placenta of patients with TTTS has at least one artery connection between the donor and the receiver. TTTS may also be developed from superficial vascular connections on the surface of the placenta in a small number of patients 23. Therefore, the formation and development of placental blood vessels, particularly VEGF-R1 and VEGF-R2 receptors, significantly contribute to the pathogenesis of TTTS. Our study showed that maternal VEGF-R1 levels were higher in twins compared to single pregnancy and were decreased in the second trimester compared to the first trimester. In addition, in patients with TTTS, the concentrations of VEGF-R1 and VEGF-R2 were both decreased after surgical treatment compared to those before surgery. These results indicated that VEGF-R1 plays an important role during the development of the placental and fetal vascular systems, and that surgical treatment affects the development of placental and fetal vascular system.

A study measured the concentrations of angiogenic and anti-angiogenic factors and showed that twin pregnant women with TTTS had higher levels of Endoglin and VEGFR-1 compared to those without TTTS while the placental growth factor (PlGF) levels were lower in the group with TTTS compared to those without TTTS 24. In line with this previous study, our results also indicated that twin pregnant women with TTTS have increased VEGF-R1 levels, but only in the second trimester. In addition, VEGF-R1 mRNA has been shown to overexpress in the chorionic villi of the donor but not in those of the recipient. Some TTTS cases may present with hypoxia or ischemia in the donor villi, which lead to the hypoxia/ischemia in donor villi due to the decreased fetal villi perfusion rather than the abnormality of placenta circulation 25. The expression of VEGF-R1 has been reported to be inversely correlated with fetal weight at birth and Apgar score in patients with gestational hypertension 26. Our results showed a significant decrease in VEGF-R1 and VEGF-R2 levels in twin pregnant women with TTTS after being treated with laser surgery suggesting a role of VEGF-R1 and VEGF-R2 in assessing the effectiveness of TTTS treatment. These results also suggest that the surgical treatment with laser affects the formation and regeneration of vessels in pregnant women with TTTS. Furthermore, in twins with TTTS, higher concentrations of natriuretic peptide and endothelin-1 have been observed in recipients compared to donors, and have been associated with cardiac dysfunction in the receiving fetus 27. Another study observed that the donors have lower leptin levels and growth factors such as insulin-II in umbilical cord blood compared to recipients in twins with TTTS 28. This may be the cause of the placenta heterogeneous metabolic function that leads to the growth restriction in those twins.

In our study group, we could not detect IL-1β and IL-8, nevertheless, IL-6 could be detected in both twin pregnant women with and without TTTS. Although no significant difference in IL-6 levels between the two groups of monochorionic twin pregnant women in the first trimester was observed, IL-6 levels seem to be increased in twin pregnant women with TTTS compared to those without TTTS in the second trimester. In addition, in twin pregnant women with TTTS treated with laser surgery, IL-6 levels were significantly increased after surgery compared to those before surgery. This result suggests that IL-6 levels are involved in the pathogenesis of TTTS and can be used as a marker for the surgical treatment of TTTS.

The cytokines from Th1 cells involved in inflammatory responses include interferons, TNF-α, and interleukins (IL-1β, IL-2, IL-6, and IL-12) 29. Cytokines have been detected in the placental and fetal membrane of normal pregnancy, and have also been associated with homeostasis and maternal placental function 18. Inflammatory interleukins such as IL-6 and IL-1β produced by oocytes and fetal membranes have been associated with spontaneous preterm delivery and infections in pregnant women 30. However, IL-6 is the only cytokine that is directly associated with TTTS 31. IL-6, IL-1β, TNF-α, IL-10, IL-4, IL-8 and TIMP-1 levels are higher in the amniotic fluid than in plasma suggesting that the abnormality of cytokine expression in pregnant women with TTTS by laser as local cytokine release. In addition, no significant difference in the plasma and amniotic laser-mediated intoxicating cytokine concentrations of the patients except for the decrease in epidermal growth factor in the amniotic fluid after treatment compared to those before treatment 32. Although IL-8 levels are not detected in our study subjects, IL-6 levels are increased in twin pregnant women with TTTS after surgical treatment suggesting a role of IL-6 in response to the treatment, and thus that IL-6 may serve as a marker for the prognosis of surgical treatment in twin pregnant women with TTTS. A limitation of the current study is the small sample size, therefore, evaluation of interleukin expression levels in twin pregnant women with TTTS in larger sample sizes is needed to further understand the functional role of interleukin in patients with TTTS.

## Conclusion

Our study showed that soluble VEGF-R1 levels are higher in monochorionic twin pregnancies with and without TTTS compared to single pregnancy and are modulated during pregnancy. Soluble VEGF-R1 and VEGF-R2 levels are significantly decreased while IL-6 levels are increased after surgical treatment with laser in twin pregnant women with TTTS. IL-6, VEGF-R1 and VEGF-R2 may be associated with the pathogenesis of TTTS and may play a prognostic role in the surgical treatment of TTTS. Further studies are required to understand the role of IL-6, VEGF-R1 and VEGF-R2 in the development of TTTS.

## Figures and Tables

**Table 1:** Baseline Characteristics of twin pregnant women with and without TTTS

Characteristics	With TTTS (n=32)	Without TTTS (n=49)	Single pregnancy (n=30)	*P* value
Age (years)	29.5 (20-38)	29 (20-36)	27 (21-43)	NS
Weight (Kg)	52.5 (42-67)	52 (48-64)	50 (37-65)	NS
Rhesus (Rh) Positive	32/32	49/49	30/30	NA
WBC (x10^6^/ml)	11.1 (7-14.4)	9.85 (7-15)	9.15 (5.2-13.6)	<0.05
Lymphocytes (%)	13.9 (7.8-27.7)	18.65 (11-29.5)	19.7 (9.5-36.7)	<0.05
Neutrophile (%)	76.7 (60.4-85.8)	70.1 (5.3-84.6)	73.35 (57.2-82.3)	NS
RBC (x10^3^/ml)	3.8 (3.24.8)	3.93 (3.22-5.65)	4.21 (3.54-4.73)	NS
Hemoglobin (g/l)	116.5 (96-141)	118.5 (98-135)	124.5 (92-139)	NS
HCT (%)	35 (29.9-42.9)	35.2 (29.5-40.9)	37 (29.5-39.8)	NS
PLT (x10^3^/ml)	257.5 (158-353)	236.5 (81-312)	243.5 (158-361)	NS
Fibrinogen (g/L)	4.6 (3.5-6.6)	4.05 (2.96-5.99)	3.79 (1.44-5.29)	NS
INR	0.97 (0.9-1.06)	0.98 (0.92-1.1)	1.03 (0.95-1.16)	NS
Prothrombin Time (PT)	104 (91-118)	102 (88-114)	111 (100.9-126)	NS
Glucose (mg/l)	4.9 (3.36-7.95)	4.7 (3.12-7.95)	4.6 (3.8-6.3)	NS
Urea (mmol/l)	2.6 (1.13-4.55)	2.74 (1.43-3.7)	2.87 (1.6-4.52)	NS
Creatinine (mmol/l)	54.1 (25.4-69.7)	56.5 (46.5-70.2)	56.9 (45.5-78.7)	NS
ALT (IU/ml)	22.8 (6.1-207.2)	17.9 (5.85-149.7)	14.36 (8.2-113.5)	<0.05
AST (IU/ml)	25.2 (13.86-151.9)	21.4 (16.2-82.8)	21.25 (14.4-89.9)	<0.05
Cholesterol (mmol/L)	6.3 (3.7-9.6)	4.66 (3.56-7.08)	4.4 (3.2-5.95)	NS
Albumin (g/L)	34.8 (29.4-39)	36 (3.46-40.7)	39.4 (35.1-44.35)	NS

**Abbreviation**: AST and ALT, aspartate and alanine aminotransferase; WBC, white blood cells; RBC, red blood cells; PLT, platelets; HCT, Hematocrits; INR, International Normalized Ratio; NS, not significant; NA, not applicable. *P* values were calculated using the Kruskal Wallis test.
